# MRIP Regulates the Myosin IIA Activity and DDR1 Function to Enable Collagen Tractional Remodeling

**DOI:** 10.3390/cells9071672

**Published:** 2020-07-11

**Authors:** Nuno M. Coelho, Andrew Wang, Petar Petrovic, Yongqiang Wang, Wilson Lee, Christopher A. McCulloch

**Affiliations:** Faculty of Dentistry, University of Toronto, Toronto, ON M5G 1G6, Canada; ayb.wang@mail.utoronto.ca (A.W.); p.petrovic3@gmail.com (P.P.); yongqiang.wang@utoronto.ca (Y.W.); wilson.lee@utoronto.ca (W.L.); christopher.mcculloch@utoronto.ca (C.A.M.)

**Keywords:** MRIP, DDR1, non-muscle myosin IIA, collagen remodeling

## Abstract

DDR1 is a collagen adhesion-mechanoreceptor expressed in fibrotic lesions. DDR1 mediates non-muscle myosin IIA (NMIIA)-dependent collagen remodeling. We discovered that the myosin phosphatase Rho-interacting protein (MRIP), is enriched in DDR1-NMIIA adhesions on collagen. MRIP regulates RhoA- and myosin phosphatase-dependent myosin activity. We hypothesized that MRIP regulates DDR1-NMIIA interactions to enable cell migration and collagen tractional remodeling. After deletion of MRIP in β1-integrin null cells expressing DDR1, in vitro wound closure, collagen realignment, and contraction were reduced. Cells expressing DDR1 and MRIP formed larger and more abundant DDR1 clusters on collagen than cells cultured on fibronectin or cells expressing DDR1 but null for MRIP or cells expressing a non-activating DDR1 mutant. Deletion of MRIP reduced DDR1 autophosphorylation and blocked myosin light chain-dependent contraction. Deletion of MRIP did not disrupt the association of DDR1 with NMIIA. We conclude that MRIP regulates NMIIA-dependent DDR1 cluster growth and activation. Accordingly, MRIP may provide a novel drug target for dysfunctional DDR1-related collagen tractional remodeling in fibrosis.

## 1. Introduction

The structure and mechanical integrity of healthy connective tissues is maintained by tightly regulated remodeling processes. Remodeling comprises the synthesis, degradation, orientation and cross-linking of collagen fibrils. Dysregulated collagen remodeling includes the formation of highly compacted and cross-linked collagen fibers. These processes result in local matrix stiffening, which is associated with organ fibrosis and tumor stroma [1,2,3]. At the core of collagen remodeling processes are trans-membrane mechanoreceptors. These receptors bind tightly to collagen fibers and enable the transmission of actomyosin-generated contraction forces to collagen fibrils. The integrins α2β1, α11β1 and the discoidin domain receptors (DDR 1 and 2) are widely expressed fibrillar collagen adhesion receptors in mammals. Integrins have long been considered as the “classical” mechano-receptors and signal transducers. There is much less known about their interactions with fibrillar collagen in tractional remodeling [4,5,6]. In contrast, there is little definitive information on the role of DDRs, which is a family of receptor tyrosine kinases (RTK). DDRs exhibit an unusually slow collagen-induced activation mechanism. This mechanism is in marked contrast to the rapid activation kinetics of classical RTKs [7,8].

The detailed mechanism of DDR1 activation is not well-understood. Recently we and others identified a link between collagen-induced DDR1 clustering and the activation of the cytoplasmic kinase domain of DDR1 by phosphorylation of tyrosine 792 (Y792). This process then leads to phosphorylation of Y513 in neighboring dimers [9,10]. We found that cells expressing kinase-depleted DDR1d, or cells expressing kinase-dead DDR1e, or cells expressing DDR1b but cultured on non-activating fibronectin substrates, formed fewer and smaller clusters compared with cells expressing full-length kinase active DDR1b that were cultured on collagen. We considered that collagen-induced DDR1 clustering induces DDR1 activation. This activation reinforces DDR1 binding to collagen that may involve a feedforward mechanism that increases receptor clustering [10]. Initial DDR1 clustering is not sufficient for DDR1 activation and DDR1 kinase activity is only triggered when DDR1 aggregates into dense clusters [11]. These observations support our proposed model and could explain the slow kinetics of DDR1 activation after binding to collagen.

We also found that collagen-induced DDR1 activation results in increased actomyosin contractility. After binding to collagen, clustering of DDR1 promotes activation of its kinase domain. This activation enhances the association of DDR1 with non-muscle myosin IIA (NMIIA) filaments that in turn are activated by the myosin light chain (MLC) kinase. This process generates a positive feedback loop. In turn, the feedback loop strengthens cell adhesion and optimizes the transmission of NMIIA-dependent contractile forces to collagen fibrils [10,12]. Despite these advances, the mechanisms that regulate the transmission of NMIIA-generated forces through DDR1 are not defined.

NMIIA is an abundant contractile protein in cell adhesion complexes [13,14]. NMIIA can bind a wide variety of protein. Some of these proteins modulate its contractile activity and thereby strongly influence collagen remodeling [15,16,17]. NMIIA activity is controlled by the phosphorylation of the MLC at Ser 19. Phosphorylation of Ser 19 and Thr 18 leads to a conformational change of NMIIA from an inactive, folded molecule to an active, extended molecule. This activation enables myosin filament assembly and tight control of the ATPase activity of the NMIIA motor domain [18,19]. The two main kinases involved in the phosphorylation of Ser 19 are MLCK and Rho-associated kinase (ROCK). The activity of ROCK is controlled by members of the Rho family of small GTPases, RhoA, RhoB, and RhoC. These proteins play critical roles in the regulation of stress fiber assembly and contractility. Both Ser 19 and Thr 18 are dephosphorylated by myosin light chain phosphatase (MLCP) to terminate ATPase activity, which thereby enables the generation of a contraction cycle [20,21]. The myosin phosphatase Rho-interacting protein (MRIP) is a scaffold protein that binds actin filaments, MLCP, and RhoA. In turn, RhoA enhances the activity of MLCP and affects the MLC of NMIIA [22,23,24].

Here we used tandem mass tagged mass spectrometry to identify MRIP as a component of an adhesion complex that involves DDR1 and NMIIA. Notably, DDR1 can regulate adhesion to collagen by modifying integrin activation [25,26]. Further, integrins regulate collagen migration and contraction through their associations with actomyosin [4]. In view of this complexity for dissecting the control systems that regulate DDR1-mediated collagen tractional remodeling, we used a specialized cell model. This model expresses DDR1 but not β1 integrin. With this model we studied DDR1 and its interactions with NMIIA in collagen remodeling, independent of integrin function. Our data provide insight into a novel regulatory system by which MRIP modulates DDR1-mediated collagen adhesion and remodeling by tractional forces. Further, we provide evidence for functional interactions between NMIIA activity, the formation of DDR1 adhesion clusters, and DDR1 tyrosine kinase activation.

## 2. Materials and Methods

### 2.1. Reagents

Anti-DDR1 (C6, sc-374618) and anti-M-RIP (C-14): sc-135494) were from Santa Cruz Biotechnology (Dallas, TX, USA). Anti-Human DDR1 and anti-Human DDR2 were from R&D Systems (Minneapolis, MN, EUA). Anti-phospho-DDR1 (Y792); anti-M-RIP (D8G8R); anti-Myosin Light Chain 2 (D18E2); and anti-Phospho-Myosin Light Chain 2 (#3675 and #3671) were from Cell Signaling Technology (Danvers, MA, EUA). Anti-IgG isotype (ab37415); anti-integrin beta 3 (EPR2342); NMIIA (2B3, ab55456); and anti-integrin beta 5 were from Abcam (Cambridge, UK). Anti-integrin beta 1; anti-β-Actin; fibronectin from human plasma; and puromycin were from Millipore Sigma (Burlington, MA, USA). Anti-non-muscle myosin (BT-564); Alexa Fluor 488 goat anti-mouse; Alexa Fluor 568 goat anti-mouse; Alexa Fluor 488 goat anti-rabbit; Alexa Fluor 568 goat anti-rabbit; Alexa Fluor 568 Donkey anti-goat; Alexa Fluor 488 phalloidin; rhodamine phalloidin; Subcloning Efficiency DH5α Competent Cells; and G 418 disulfate salt were from Thermo Fisher Scientific (Waltham, MA, USA). Nutragen, bovine collagen solution, 6 mg/mL was from Advanced BioMatrix (Carlsbad, CA, USA).

### 2.2. Cells

Integrin β1-deficient GD25 cells were provided by Reinhard Fässler (Max-Planck Institute for Biochemistry, Munich, Germany). For β1^-/-^ knockout cells, the second exon of the β1 integrin gene in embryonic stem (ES) cells was disrupted with a gene trap vector with a β-galactosidase-neomycin fusion DNA. ES cells were immortalized with recombinant retroviruses that transduced the SV-40 large T. A single clone was established that was mutated in both alleles. The homozygous mutant clone did not produce β1 integrin mRNA or protein (Fassler et al., 1995). GD25 cells were stably transfected with DDR1 (b-isoform) plasmids (GD25 OE cells) [1]. Cells were cultured in Dulbecco’s-modified Eagle’s medium (DMEM) with 10% fetal bovine serum, penicillin, and streptomycin (100 U/mL and 100 μg/mL). GD25 stably OE DDR1 required a selective growth medium supplemented with 200 μg/mL G418.

### 2.3. MRIP Deletion by CRISPR/Cas9

The MRIP knockout cell line was generated in mouse GD25 OE cells with high DDR1 expression using CRISPR/Cas9 technology by Applied StemCell (Milpitas, CA, USA). Exon 1 of mouse MRIP was mutated, leading to a frame shift of the downstream MRIP sequence, and the creation of a premature STOP codon. Briefly, a mixture of plasmids containing gRNAs targeting MRIP exon 1, and the Cas9 gene was electroporated into GD25 OE cells. The transfected cells were cultured in drug-free medium for 48 h, and then selected using 2–3 μg/mL puromycin for a period of 24 to 48 h. Surveyor assay was used to confirm the presence of insertions or deletions (indels). Single cell cloning was performed. Cell clones were genotyped by PCR and sequencing. Five KO clones were expanded. The expanded clones doubling time was similar the parental GDOE cells (14 to 16 h) confirming no impact of MRIP KO on cell viability.

### 2.4. Transfection

Takara’s In-Fusion HD cloning technology (Mountain View, CA, USA) was used for gene cloning. Briefly, a primer pair 5’-CGCCGGAATTAGATCTGCCACCATGTCGGCGGCC-3’ and 5’-ATTCGTTAACCTCGAGTTATCACTTATCGTCGTCATCCTTG-3’ was used to amplify myc-tagged mouse Mrip variant 1 (NM_201245.3) cDNA synthesized by GenScript (Piscataway, NJ, USA). The amplified cDNA was inserted to pMSCVpuro that was linearized by BglII/XhoI. The sequence of the insert was confirmed by the Centre for Applied Genomics (Sickkids, Toronto). pMSCVpuro-Mrip was co-transfected with pVSV-G to a retroviral packaging cell line GP-293 to produce retrovirus expressing Mrip. Mrip knockout cells were infected with the virus and selected for two weeks with 3 µg/mL puromycin and maintained in cell culture medium containing 1 µg/mL puromycin. The expression of Mrip was confirmed by Western blot.

### 2.5. Collagen and Fibronectin Substrates

Polymerized collage substrates were prepared from pepsin-treated, bovine dermal type I collagen. Collagen solutions were diluted to a final concentration of 1 mg/mL and neutralized with 0.1 M NaOH to pH = 7.4. Collagen solutions (~100 μL) were poured onto MatTeK 60 mm glass bottom culture dishes plasma/APTES/glutaraldehyde treated and gently covered with an untreated glass coverslip. Samples were incubated at 37 °C in 5% CO_2_ until collagen polymerization was complete (>90 min). Untreated top coverslips were gently detached from the collagen gels by addition of PBS. PBS was replaced by cell culture medium and the cells cultured for indicated periods of time. Substrates were prepared by exposure to oxygen plasma and immediate treatment with sequential 15 min incubations in 2% APTES and 0.1% v/v glutaraldehyde. For immunoblotting, immunoprecipitation, and for the fibrillar collagen films substrates on glass chamber slides, excess of neutralized 1 mg/mL collagen solution was added to tissue culture plates or to plasma treated 8-well glass chamber slides for 15 min. Collagen excess was gently removed and substrates were allowed to air dry under the hood.

Fibronectin was diluted to 10 μg/mL in 1× PBS; applied to tissue culture plates or 8-well glass chamber slides; and incubated at 37 °C for 30 min before wash with 1× PBS.

### 2.6. Immunofluorescence and Microscopy Analysis 

Cells cultured on different collagen and or fibronectin substrates were fixed with 4% PFA for 10 min, when indicated permeabilized in 0.3% Triton X-100 for 5 min and blocked with 1% BSA and/or 5% goat serum. Samples were incubated with primary antibodies for 1 h at 37 °C, washed, and incubated with appropriate secondary antibody for 45 min at RT. In some experiments, cells were labeled with fluorescein or rhodamine phalloidin. Images were obtained with a Leica TCS confocal microscope (Mannheim, Germany). Collagen fibers were visualized using confocal reflectance microscopy. Analysis and quantification of acquired images was done with Image J or FIJI. 

Fibrillar collagen images were quantified to provide estimates of collagen remodeling activity using 2 approaches: compaction (fibril intensities) and reorganization (alignment index) of collagen fibers around cell extensions. For measuring collagen compaction, the intensity of the confocal reflectance image of collagen fibrils in fixed areas was computed around cell extensions. Measurements from each sample were normalized against fluorescence intensities in samples without cells. Local collagen fiber alignment was quantified in regions of interest in the fixed areas around cell extensions using fast Fourier transform (FFT) and Oval Profile (ImageJ plug-in) as described earlier [2]. The alignment index was defined based on higher pixel intensities in a specific angle, which was related to the orientation of collagen fibers in the corresponding direction. Quantification was done by calculation of the area under the intensity curve within ± 10 degrees of the peak using an in-house written in C++ code.

Pearson coefficients were determined with colocalization 2 plug-in in Fiji (http://fiji.sc/Coloc_2). The number and area of DDR1 adhesions were quantified as described elsewhere [2] from fluorescent images acquired on the confocal microscope with a 63× objective. Briefly, in image J the image background was subtracted using the sliding paraboloid (radius set to 50 pixels), the local image contrast was enhanced with CLACHE plug-in (block size = 19, histogram bins = 256, maximum slope = 6, no mask, and fast), background minimized with mathematical exponential (EXP), brightness and contrast adjusted automatically, image filtered with the LOG3D plug-in (sigmaX = 5 and sigmaY = 5), followed by auto threshold. Number and area of DDR1 adhesions were obtained with Analyze Particles command (size = 50-infinity and circularity = 0.00–0.99). 

### 2.7. Immunoblotting

Equal amounts of protein were resolved by SDS/PAGE and transferred to nitrocellulose membranes. Immunoblotting was performed by blocking membranes with 5% BSA in TBS for 1 h followed by overnight incubation with primary antibodies at 4 °C in 2.5% BSA in TBS with 0.1% Tween-20. Membranes were incubated with respective secondary antibody for 1 h in TBS with 0.1% Tween-20 at room temperature. Image Studio from LI-COR (Lincoln, NE, USA) was used for detection and analysis of immunoblots.

### 2.8. Immunoprecipitation

Cells were lysed in 1% Tris-NaCl-Triton immunoprecipitation buffer (20 mM Tris, pH 7.5, 1% Triton X-100, 0.1% SDS, 150 mM CaCl) containing 1 mM phenylmethylsulfonylfluoride, 1 mM Na_3_VO_4_ 10 μg/mL leupeptin and 10 μg/mL aprotinin. Equal amounts of protein from cleared extracts were immunoprecipitated with Dyna-beads Protein G from life technologies (Carlsbad, CA, USA) according to the manufacturer’s protocol.

### 2.9. Tandem Mass Tagged Mass Spectrometry

The accession number for mass spectrometry proteomics data reported in this paper is PRIDE: PXD005722. DDR1 adhesion complexes preparation, processing, extraction, and analysis by MS/MS were previously described [2] using a C-terminal anti-DDR1 antibody bound to superparamagnetic DynaBeads. Briefly, GD25 WT and GD25 OE cells were plated on either collagen or fibronectin for one or eight hours prior to lysis and immunoprecipitation. Samples were reduced, alkylated, digested, and TMT-labeled according to the manufacturer’s directions (Thermo Fisher TMT 10 Plex, Waltham, MA, EUA). Samples were analyzed on an Orbitrap analyzer (Q-Exactive, Thermo Fisher) outfitted with a nanospray source and EASY-nLC nano-LC system (Thermo Fisher, Waltham, MA, EUA). Lyophilized peptide mixtures were dissolved in 0.1% formic acid and loaded onto a 75 μm × 50 cm PepMax RSLC EASY-Spray column filled with 2 μM C18 beads (Thermo Fisher, Waltham, MA, EUA). Peptides were eluted using formic acid acetonitrile. Peptides were introduced by nano-electrospray into the Q-Exactive mass spectrometer. Tandem mass spectra were extracted, and all MS/MS samples were analyzed using Sequest (Thermo Fisher Scientific; v. 1.4.1.14) and X! Tandem (The GPM, thegpm.org; v. CYCLONE (2010.12.01.1)). Sequest was set up to search Uniprot_Mouse_Nov_18_2015.fasta (Downloaded November 18 2015, 74,993 entries) assuming the digestion enzyme trypsin. X! Tandem was set up to search the Uniprot_Mouse_Nov_18_2015 database. Scaffold (v. Scaffold_4.7.5, Proteome Software Inc., Portland, OR, USA) was used to validate MS/MS based peptide and protein identifications.

### 2.10. Wound Healing Assay

Different cells were used in wound (2D) migration assays using 2-well culture-inserts from ibidi GmbH (Gräfelfing, Germany). Briefly, 24-well tissue culture plates were coated with either 10 µg/mL fibronectin or with 1 mg/mL fibrillar collagen. After coating the culture-inserts were added to each well and 1 × 10^5^ cells were plated in each well. After appropriate culture time to reach confluency a cell-free gap of 500 µm was created by removal of the culture-insert. Experiments were performed on an environmentally controlled microscope stage, Axiovert 135, from ZEISS (Oberkochen, Germany), and images were acquired every 2 h up to 24 h using a phase-contrast objective (10×). The percent of wound closure in different fields was calculated with Image J software.

### 2.11. Collagen Substrate Deformation by Cell-Generated Forces

Collagen solutions were mixed with paramagnetic beads (2 µm diameter), which served as fiduciary markers for tracking the deformation of the fibrillar collagen under adherent cells. The relatively greater mass of the magnetite beads enabled settling of the beads towards the bottom surface of the gel prior to polymerization. Gels were inverted after collagen polymerization was complete. The bead displacement around each cell was determined from a time series of phase contrast images that were collected using a phase-contrast microscope (Zeiss Axiovert 135) in environmentally controlled conditions (37 °C/5% CO_2_). The initial bead position was determined 1 h after cell seeding, when cells were properly attached to the gel surface. A time series of 120 images was collected at a frequency of one image every 5 min. Image stacks were aligned using Image J plug-in Linear Stack Alignment with scale-invariant feature transform (https://imagej.net/Linear_Stack_Alignment_with_SIFT). All images were then compared one-by-one to the first image using the Particle Image Velocimetry PIV plug-in in ImageJ (https://sites.google.com/site/qingzongtseng/piv#tuto) to map bead displacement over time [27]. The magnitude of the displacement vectors obtained from the PIV (mag1) plug-in was summed in order to calculate the total collagen substrate deformation (i.e., the deformation magnitude) for each cell type.

### 2.12. RhoA Activation Assay

RhoA activity assay was performed according to the manufacturer’s instructions for a RhoA activation assay Biochem Kit from Cytoskeleton Inc (Denver, CO, USA). Cells were lysed in immunoprecipitation buffer. GTP-bound RhoA was immunoprecipitated from cleared lysate with glutathione S-transferase-tagged Rhotekin-Rho-binding domain protein bound to glutathione agarose. The beads were washed and the immunoprecipitates were analyzed by Western blot and probed with RhoA-specific monoclonal antibody from Santa Cruz Biotechnology (Dallas, TX, USA). The total cell lysate (40 μg) was also probed for RhoA to ensure equality across conditions.

### 2.13. Isolation of DDR1 Adhesion Complexes

DDR1 adhesion complexes were prepared from cells incubated with collagen coated magnetite beads after 1 and 2 h of incubation as previously described for focal adhesions isolation [3]. Briefly, cell-bead complexes were scraped into ice-cold cytoskeleton extraction buffer (0.5% Triton X-100, 50 mM NaCl, 300 mM sucrose, 3 mM MgCl_2_, 20 mg/mL aprotinin, 1 mg/mL leupeptin, 1 mg/mL pepstatin, 1 mM PMSF, and 10 mM PIPES, at pH 6.8). After washing beads 3 times with CSKB using magnetic separation to remove non-specifically bound proteins, the remaining bead-associated proteins were eluted in Laemmli sample buffer by boiling for 10 min.

### 2.14. Fluorescence Resonance Energy Transfer

Acceptor Photobleaching - Fluorescence fluorescence resonance energy transfer (AP-FRET) was performed on a Leica SP8 confocal microscope with the Leica AP-FRET wizard. Images were acquired with a 40× NA 1.3 oil objective at 512 × 512-pixel resolution. Photobleaching was minimized during acquisition by using low laser power (10% for 405 nm and 488 nm). A region of interest (ROI; 1 × 1 μm) was chosen in selected areas and 85% laser power was used with three iterations for photobleaching the acceptor DDR1-YFP. Pre-bleach and post-bleach images were acquired using the same settings. The efficiency of energy transfer (E%) was calculated using the equation: E% = (donor post-bleach − donor pre-bleach) × 100/ (donor post-bleach).

### 2.15. Fluorescence Recovery after Photobleaching

FRAP was performed using the FRAP module on a Leica TCS SP8 confocal microscope and a 63×/1.4 NA oil immersion objective and a 488 nm argon laser. Cells transiently transfected with DDR1b-YFP constructs were cultured for 6 h on either collagen or fibronectin and before FRAT experiments. In FRAP experiments 1 μm^2^ ROI (region of interest) was photobleached encompassing the target DDR1 clusters and its surrounding neighbors and fluorescent recovery was monitored for 150 s after. Images were analyzed with FIJI software. To compensate for cell drift, the Linear Stack Alignment with SIFT plug-in was used. Fluorescence value measurements were then exported to Microsoft Excel where they were normalized and scaled between 0–1 using:

(1) $$I_{\mathrm{norm}}=\frac{\left[\left(I_{\mathrm{bleach}}-I_{\mathrm{nonbleac}}\right)n\;-\;\max\left(I_{\mathrm{bleach}}-I_{\mathrm{nonbleach}}\right)\right]}{\left[\max\left(I_{\mathrm{bleach}}-I_{\mathrm{nonbleach}}\right)-\min\left(I_{\mathrm{bleach}}-I_{\mathrm{nonbleach}}\right)\right]}$$

Subsequently, these Excel files containing the normalized values of fluorescence recovery were imported to GraphPad Prism (San Diego, CA, USA), to perform curve fitting analysis, plot, and do statistical analysis of the various parameters obtained from the double exponential equation used for the fit.

### 2.16. Statistical Analysis

All continuous variables are presented as mean ± SEM of at least three independent experiments. Statistical significance (*p* < 0.05) was determined using unpaired t-test or analysis of variance (ANOVA) as appropriate and performed with GraphPad Prism software (San Diego, CA, USA).

## 3. Results

### 3.1. MRIP is Enriched in DDR1-Collagen-Adhesion Complexes

**Figure 1:**
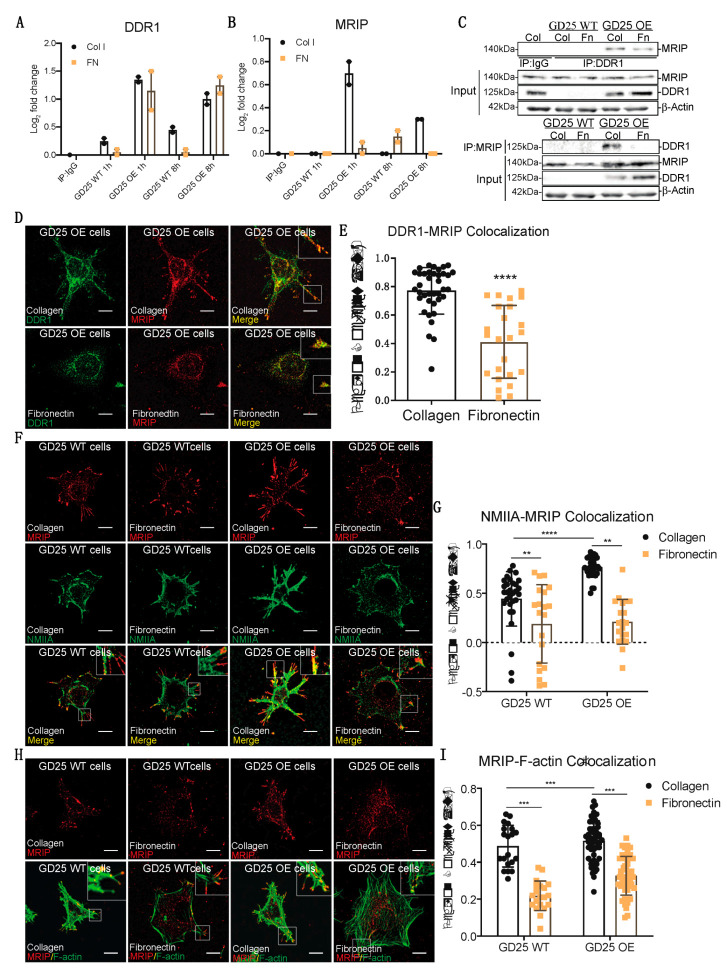
MRIP is enriched DDR1 collagen adhesion complexes. (**A**,**B**) DDR1 (C-20: sc-532) was immunoprecipitated from cell lysates of GD25 WT and GD25 OE cells cultured on collagen or fibronectin for 1 or 8 h. Immunoglobulin G (IgG, ab37415) was immunoprecipitated from cell lysates of GD25 OE cells cultured on collagen for 8 h and used as control. Immunoprecipitates were processed and analyzed by liquid chromatography - tandem mass spectrometry (LC-MS/MS). Plot represents the relative abundance of DDR1 (A) and MRIP (B) in each immunoprecipitate, *n* = 2. (**C**) IgG, DDR1 (C-6: sc-374618), and MRIP (D8G8R, CS), were immunoprecipitated from cell lysates of cells cultured on fibrillar collagen or fibronectin. The immunoprecipitates were immunoblotted for DDR1 and MRIP. Whole-cell lysates were immunoblotted for β-actin as loading control. (**D**,**F**,**H**) Indicated cells were cultured on collagen or on fibronectin for 3 h and co-immunostained for MRIP (C-14: sc-135494, red) and DDR1 (C-6: sc-374618, green), for MRIP and NMIIA (2B3, ab55456, green), and for MRIP and F-actin (green). (**E**,**G**,**I**) Pearson coefficients were obtained by quantification of the fluorescent images using the colocalization2 plug-in in Fiji. Data are reported as mean ± SD, *n* = 3, at least 20 cells per group in E, G, and I. ** *p* < 0.005, *** *p* < 0.0005, **** *p* < 0.0001. Scale bar, 10 µm.

**Figure 2:**
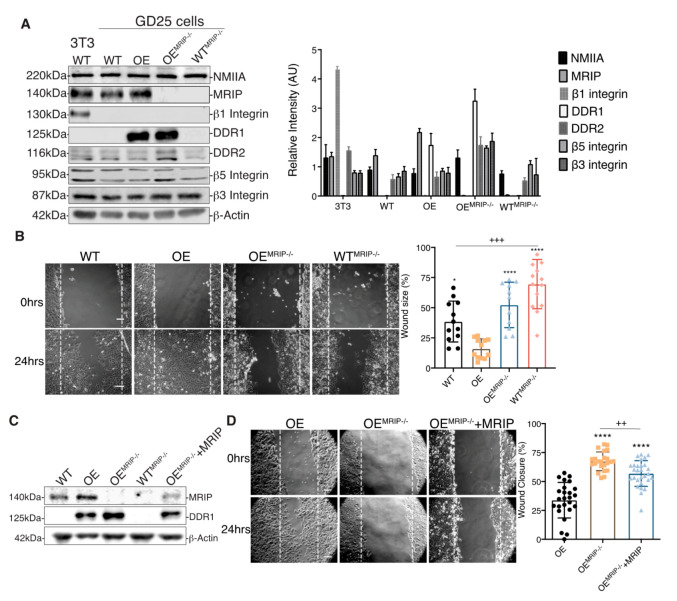
MRIP deletion inhibits cell migration on collagen. (**A**) Immunoblots for evaluation of indicated protein expression levels in whole cell lysates of NIH 3T3 WT, GD25- WT, OE, OE^MRIP-/-^, and WT^MRIP-/-^ cells. The plot on the right represents the ratio of indicated proteins to β-actin levels for the in vitro cell model. (**B**) Wound-healing assay of confluent monolayers of WT, OE, OE^MRIP-/-^, and WT^MRIP-/-^ cells cultured on collagen and record at 0, 4, 8, and 24 h post-wounding (B, 0 and 24 h) and quantified in the plot on the right. (**C**) By Western blot we found an approximately 25% restoration of MRIP expression in OE^MRIP-/-^ cells stably transfected with mouse MRIP variant 1 (OE^MRIP-/-^+MRIP). The indicated cell lysates were also immunoblotted for DDR1 and for β-actin. (**D**) Wound-healing assay of confluent monolayers of OE, OE^MRIP-/-^, and OE^MRIP-/-^+MRIP cells cultured on collagen. Wound closure was recorded every hour for 24 h after wounding. Quantification is shown in the plot on the right. Data are reported as mean ± SD, *n* = 3, * *p* < 0.05, ^++^ *p* < 0.005, ^+++^ *p* < 0.0005, **** *p*<0.0001. Scale bar, 10 µm.

While a role for DDR1 in mechanical remodeling of collagen fibrils through its association with NMIIA has been described [2], the regulation of this association and its impact on collagen remodeling is not defined. Parental β1 integrin null GD25 WT cells. A continuous cell line was derived from an embryonic stem cell line (G201). These cells were derived from β1 integrin null mice. GD25 cells are fibroblast-like cells that express very low levels of DDR1 (Figure 1A, [2]). We stably overexpressed DDR1b in GD25 cells (GD25 OE). The GD25 WT and OE cells were cultured on collagen or fibronectin (a non-binding DDR1 ligand control) for 1 or 8 h. DDR1 immunoprecipitates from cell lysates were processed and analyzed by tandem mass tag mass spectrometry. We quantified the relative abundance of proteins that associate with DDR1. There was increased (up to 3-fold) abundance of MRIP in DDR1 immunoprecipitates when GD25 OE cells were cultured on collagen compared with cells cultured on fibronectin or with GD25 WT cells cultured either on collagen or fibronectin (Figure 1B). Immunoprecipitation of lysates prepared from cells cultured on collagen or fibronectin showed that the association of DDR1 with MRIP is enhanced specifically by collagen binding (Figure 1C).

We examined the spatial relationship of MRIP and DDR1 in GD25 OE cells. These cells do not express β1-integrin [4]. This integrin subunit is present in all collagen-binding integrins [5]. Consistent with the immunoprecipitation data (Figure 1C), there was 30% more colocalization between MRIP and DDR1 on cells cultured on collagen than on fibronectin; these proteins colocalized at the tips of cell extensions (*p* < 0.0001; Figure 1D,E). As MRIP binds to actin filaments and the regulatory myosin-binding subunit of myosin II phosphatase in vitro [6], we examined the spatial relationship of MRIP with actin filaments and with NMIIA in GD25 WT or OE cells. There was a >2-fold more colocalization of MRIP with NMIIA and with actin filaments when cells were cultured on collagen compared with fibronectin (Figure 1F-I). These data indicate that MRIP spatially associates with DDR1 and NMIIA in collagen adhesion complexes.

### 3.2. MRIP Expression Affects Cell Migration and Collagen Tractional Remodeling

The association of DDR1 with NMIIA depends on the phosphorylation of the MLC 2. As MRIP is an indirect inhibitor of pMLC [7,8], we examined the role of MRIP in the function of DDR1-adhesion complexes in β1-integrin null GD25 cells [4]. The effect of MRIP on the regulation of DDR1-NMIIA association was examined in a MRIP KO cell line generated from GD25 WT and OE parental cell lines using CRISPR-Cas9 technology (WT^MRIP-/-^ and OE^MRIP-/-^ cells, Figure 2A). The expression levels of NMIIA, DDR2, β5 Integrin, and β3 Integrin were similar for all of these cell types and for the NIH 3T3 WT cells that were used as positive control for β1 integrin-expressing cells (Figure 2A). 

The association between DDR1 and NMIIA is implicated in cell migration on collagen [9]. Accordingly we evaluated the impact of MRIP expression on cell migration on collagen using in vitro wound closure assays. Deletion of MRIP blocked the ability of cells to migrate on collagen (Figure 2B). The most rapid wound closure occurred in cells expressing DDR1 and MRIP (OE, Figure 2B). Stable transfection of mouse MRIP in OE^MRIP-/-^ cells resulted in 25% rescue of MRIP protein expression as detected by immunoblot (OE^MRIP-/-^+MRIP, Figure 2C). Analysis of cell migration showed reduced wound size for OE^MRIP-/-^+MRIP cells compared with OE^MRIP-/-^ cells (Figure 2D).

**Figure 3:**
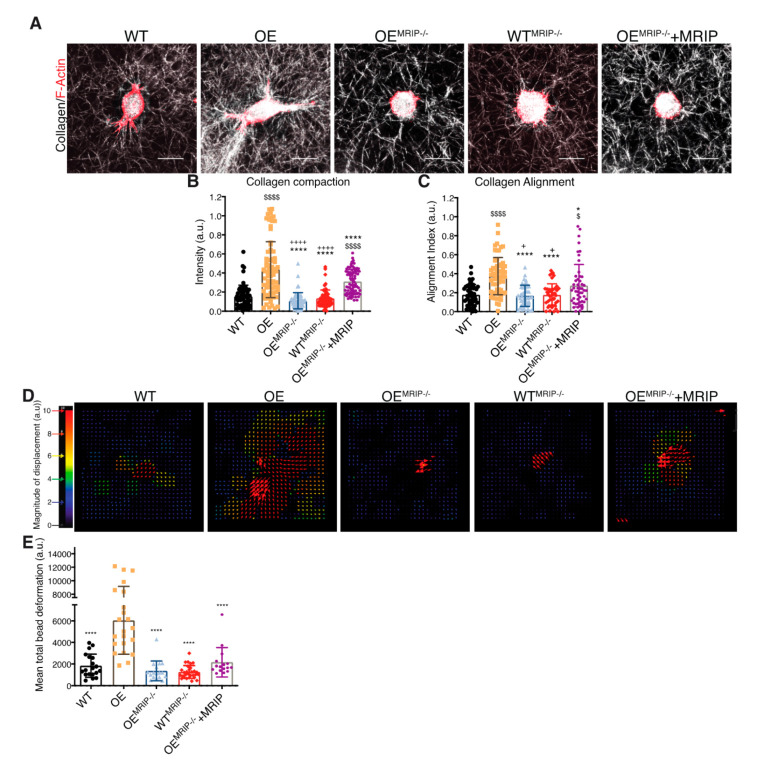
MRIP knockout reduces traction forces and collagen remodeling. (**A**) Representative images of cells cultured on fibrillar collagen gels for 24 h to evaluate and quantify collagen compaction (mean intensity of reflectance signal (**B**)), and fiber alignment (**C**). In panel (**D)**, collagen tractional remodeling was quantified by analyzing position changes of marker beads embedded in collagen gels over a 10 h sampling period. (**E**) Bead displacement was used to calculate mean cell-induced deformations by particle image velocimetry, which are displayed as vectors with color-coded magnitude as indicated in D. Data are reported as mean ± SD, *n* = 3. ^$^, ^+^ *p* < 0.05; ^$$$$^, ^++++^, **** *p* < 0.0001; ^$^ compared with WT; ^*^ compared with OE; ^+^ compared with OE^MRIP-/-^+MRIP. Scale bar, 20 µm.

We quantified the contribution of MRIP to DDR1-dependent collagen remodeling by cell-mediated traction forces in cells cultured for 24 h on fibrillar collagen gels (Figure 3A). Collagen compaction was quantified by measurements of fibril intensities in confocal reflectance light microscopy images (Figure 3B). Collagen alignment was measured in fixed regions in the cell periphery and normalized to fixed region size values in gels without cells (Figure 3C). GD25 OE cells compacted and aligned more collagen than GD25 WT, OE^MRIP-/-^, WT^MRIP-/-^, and OE^MRIP-/-^+MRIP cells. The GD25 OE^MRIP-/-^+MRIP cells compacted and aligned collagen by >3-fold more (*p* < 0.001) than GD25 OE^MRIP-/-^ and WT^MRIP-/-^ cells (Figure 3A–C).

We also examined the contribution of MRIP to DDR1-dependent collagen tractional remodeling by seeding cells at low density into three-dimensional (3D) collagen gels (1 mg/mL) containing embedded fiduciary position markers (Figure 3D). Collagen substrate deformation was calculated from bead displacements (Figure 3E) using particle image velocimetry (PIV) for 0–10 h after cell attachment [28,29]. Analysis of the resultant heat-color-coded vector map showed maximum collagen substrate deformations near the cell centroid. GD25 OE cells applied 3-fold (*p* < 0.0001) higher traction strain than MRIP null cells or cells expressing low levels of DDR1 (GD25 WT, Figure 3D and E). Re-expression of MRIP in OE^MRIP-/-^ cells (OE^MRIP-/-^+MRIP) induced marked increase of bead deformation compared with OE^MRIP-/-^ cells (Figure 3E). These results demonstrate that MRIP is important for DDR1-dependent cell migration on collagen and DDR1-NMIIA dependent collagen remodeling.

### 3.3. MRIP Enables DDR1 Cluster Formation and Growth and Stabilizes DDR1 Activation

**Figure 4:**
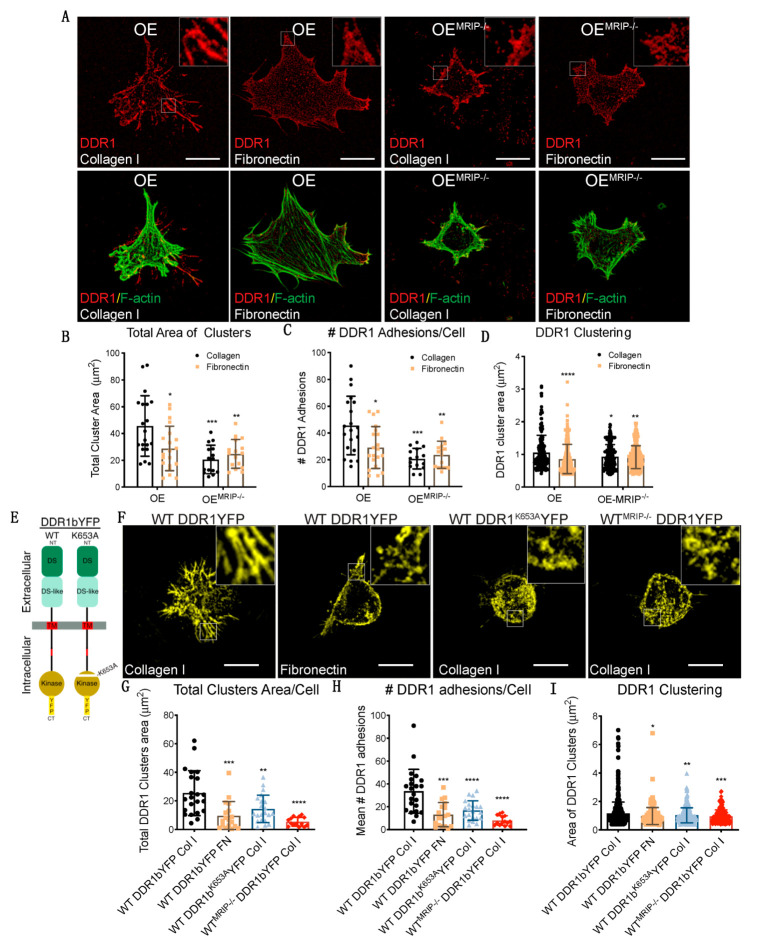
MRIP enables DDR1 cluster formation and growth. (**A**) Cells were cultured on fibrillar collagen films or on fibronectin for 8 h. An antibody against the extracellular N-terminal region of DDR1 (AF2396, R&D) was used to stain non-permeabilized preparations (top, red) followed by phalloidin to stain F-actin (bottom, green). Quantification of total cluster area, (**B**), number, (**C**), and single cluster area, (**D**), of DDR1 adhesions on collagen or on fibronectin using Fiji. Scale bar, 20 µm. (**E**,**F**) GD25 WT or WT^MRIP-/-^ cells were transfected with mouse DDR1b-YFP (full-length kinase-active isoform), or mouse DDR1b^K653A^-YFP and cultured on fibrillar collagen or on fibronectin as indicated. (**G**–**I**) Quantification of total cluster area, number, and single cluster area of DDR1b-YFP or DDR1b^K653A^-YFP adhesions on collagen or on fibronectin as indicated using Fiji. Data are reported as mean ± SD, *n* = 3. * *p* < 0.05, ** *p* < 0.005, *** *p* < 0.0005, **** *p* <0.0001. Scale bar, 20 µm.

DDR1 binding to collagen induces DDR1 clustering. In turn, clustering promotes activation of the DDR1 kinase domain (Y792) and the association of DDR1 with NMIIA [2,10,11]. We evaluated the contribution of MRIP to DDR1 cluster formation by immunostaining non-permeabilized preparations of GD25 OE or OE^MRIP-/-^ cells cultured on collagen or fibronectin for 8 h. Cells were immunostained with an antibody against the extracellular N-terminal domain of DDR1 (Figure 4A). Cells expressing DDR1 and MRIP were plated on collagen. These cells formed more numerous and larger DDR1 clusters compared with cells cultured on fibronectin, or OE^MRIP-/-^ cells (Figure 4A–D).

In some experiments, GD25 WT or WT^MRIP-/-^ cells were transfected with mouse DDR1b-YFP (full-length kinase-active isoform), or mouse DDR1b^K653A-^YFP (point mutation in ATP binding site, Figure 4E) and cultured on fibrillar collagen or fibronectin for 8 h. Imaging of cells plated on collagen showed 2-fold increases of the area and number of DDR1 clusters in GD25 WT DDR1b-YFP-expressing cells compared with same cells cultured on fibronectin, or cells expressing the non-activating DDR1 mutant (mouse DDR1b^k653A^-YFP), or WT^MRIP-/-^ DDR1b-YFP-expressing cells (Figure 4F–I). These data indicated that MRIP is not required for initial DDR1 cluster formation but is involved in the stabilization and growth of DDR1 into dense clusters.

**Figure 5:**
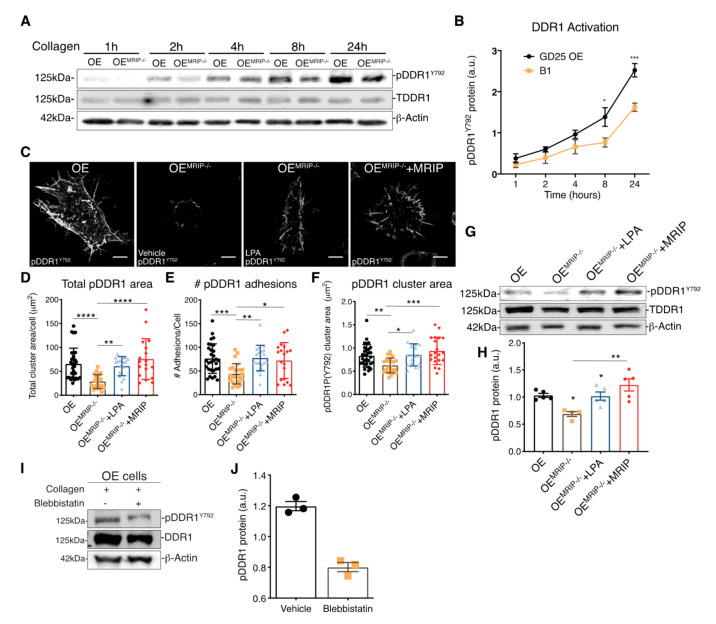
MRIP stabilizes DDR1 activation. (**A**) Cells were cultured on fibrillar collagen-coated tissue culture dishes for indicated times; whole cell lysates were immunoblotted for pDDR1^Y792^ (#11994, CS), DDR1 (C-6: sc-374618), and β-Actin. (**B**) Quantification of pDDR1^Y792^/DDR1 by densitometry of immunoblots, *n* = 3. (**C**) Cells were cultured on fibrillar collagen films for 8 h; an antibody against pDDR1^Y792^ was used to stain cell preparations. Quantification by Fiji of total cluster area, (**D**), number, (**E**), and single cluster area, (**F**), of pDDR1 ^Y792^ adhesions on collagen. Scale bar, 10 µm. (**G**) Cells were cultured on collagen for 8 h with vehicle (-) or with 20 μM lysophosphatidic acid (LPA) (+). (**H**) Quantification of pDDR1^Y792^/DDR1 by densitometry of immunoblots, *n* = 3. (**I**) Cells were cultured on collagen for 8 h with vehicle or with 25 µM of blebbistatin. (**J**) Quantification of pDDR1^Y792^/DDR1 by densitometry of immunoblots from 3 independent experiments. Data are reported as mean ± SD for D, E, F, and H and as mean ± SEM for J, *n* = 3. * *p* < 0.05, ** *p* < 0.005 *** *p* < 0.0005, **** *p* < 0.0001. Scale bar, 10 µm.

The growth of DDR1 clusters is thought to depend on a feedforward loop involving DDR1 activation [2]. Accordingly, we evaluated the effect of MRIP expression on the phosphorylation of DDR1^Y792^ in GD25 OE or OE^MRIP-/-^ cells plated on fibrillar collagen. In time-series experiments there was reduced phosphorylation of DDR1^Y792^ in OE^MRIP-/-^ cells compared with GD25 OE cells, particularly from 8 up to 24 h (Figure 5A, B). We also examined DDR1 activation by immunostaining for pDDR1^Y792^ and quantified pDDR1^Y792^ cluster area and cluster numbers in GD25 OE, OE^MRIP-/-^ or OE^MRIP-/-^ cells that re-expressed MRIP (OE^MRIP-/-^+MRIP cells) cultured on collagen for 8 h. Depletion of MRIP reduced by 2-fold the area and number of pDDR1^Y792^ clusters (Figure 5D–F). In some experiments, OE^MRIP-/-^ cells were cultured with vehicle or with lysophosphatidic acid (LPA; 20 µM) to activate Rho and ROCK and inhibit MLC phosphatase [12]. Treatment of OE^MRIP-/-^ cells with LPA or re-expression of MRIP in OE^MRIP-/-^ cells (OE^MRIP-/-^+MRIP cells) resulted in restoration of the numbers and areas of pDDR1 clusters. These data were similar to cells expressing MRIP (OE cells, Figure 5C–F). We also immunoblotted for pDDR1^Y792^ in the same cell types that had been plated on collagen. MRIP knockout reduced DDR1 activation by 40% while LPA treatment restored DDR1 activation similar to OE^MRIP-/-^ cells in which MRIP expression was restored (OE^MRIP-/-^+MRIP cells, Figure 5G, H). NMIIA activity is associated with increased phosphorylation of MLC and also with the ATPase activity of the myosin motor domain. In consideration of this, we treated GD25 OE cells with the NMIIA ATPase inhibitor blebbistatin. In cells plated on collagen, immunoblotting showed a 3-fold reduction of pDDR1^Y792^ after blebbistatin compared with vehicle (Figure 5I, J). Collectively these data indicate that MRIP-dependent NMIIA activity contributes not only to the stabilization and growth of DDR1 into dense clusters, but also perpetuates DDR1 activation.

**Figure 6:**
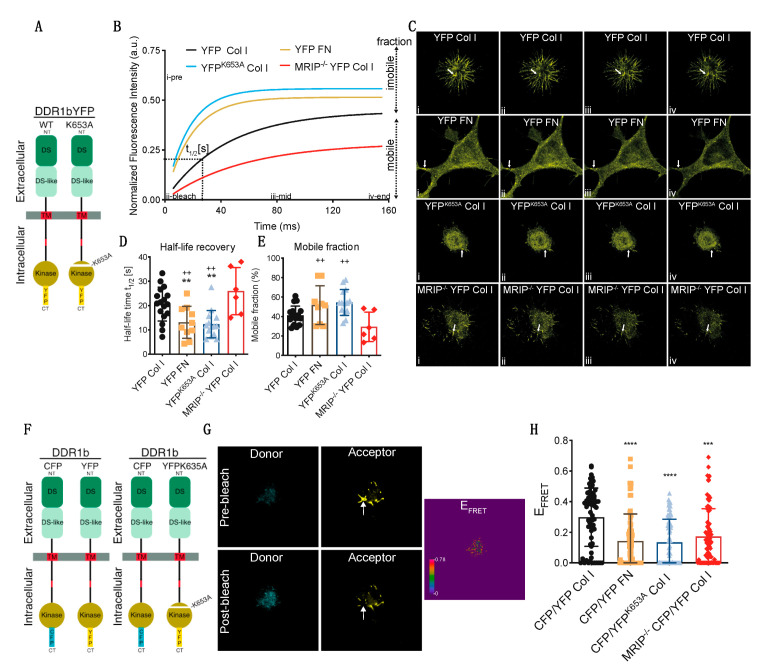
MRIP enables the feedforward loop that links DDR1 clustering and activation. (**A**) GD25 WT or WT^MRIP-/-^ were transfected either with DDR1b-YFP or DDR1b^k653A^-YFP and cultured on fibrillar collagen or fibronectin as indicated. For fluorescence recovery after photobleaching measurements, regions of interest (ROIs: 1 μm^2^ area indicated with arrow) in (**C**) containing DDR1 clusters were bleached with argon laser at 488 nm. Fluorescence in ROIs was measured before bleaching and for 150 s after bleaching. (**B**) Typical normalized FRAP curves for each condition are displayed. (**C**) Images for each condition before bleaching (i: i-pre in B), after bleach (ii: ii-bleach in **B**), at intermediate recovery point (iii: iii-mid in B), and at the end of record fluorescent recovery (iv: iv-end in B). The FRAP-half-life recovery, (**D**), and FRAP mobile fractions, (**E**), for each condition were then calculated from the normalized curves. (**F**) DDR1 clustering was detected by co-transfecting GD25 WT or WT^MRIP-/-^ cells with DDR1b-CFP (donor) and DDR1b-YFP (acceptor) probe as FRET pair. To access the contribution of DDR1 activation for cluster growth we co-transfected WT cells with the DDR1-CFP and DDR1^K653A^-YFP (YFP^K653A^) FRET probe. The cells were co-transfected with the indicated FRET pairs and cultured on collagen or fibronectin. (**G**) FRET efficiency was calculated from donor and acceptor pre- and post-bleach images (arrow indicates bleach ROI) with the acceptor-photobleaching-FRET mode in Leica SP8 confocal microscope. (**H**) Plot represents the mean FRET efficiency for interactions of indicated FRET pairs in cells in the indicated conditions. Data are reported as mean ± SD, *n* = 3. ^++^, ** *p* < 0.005, *** *p* < 0.0005, **** *p* < 0.0001.

We used fluorescence recovery after photobleaching (FRAP) analyses to examine the contribution of MRIP to DDR1 cluster growth and stabilization of DDR1 activation. GD25 WT or GD25 WT^MRIP-/-^ cells were transfected with either YFP-tagged DDR1b or YFP-tagged DDR1b^K653A^ (YFP^K653A^) and plated on collagen or fibronectin. After 6 h of culture, DDR1 clusters were photobleached and fluorescence recovery was measured for 150 s after bleaching (Figure 6A–C). Typical normalized FRAP curves for each condition are presented in Figure 6B. These data were used to calculate half-time recovery and mobile fractions from measurements before and after bleaching (shown in Figure 6B–E). Cells expressing DDR1bYFP cultured on fibronectin or cells expressing the non-activating mutant YFP^K653A^ showed 2-fold faster recovery and 25% higher mobile fractions compared with cells expressing DDR1bYFP and MRIP or cells depleted of MRIP. Cells depleted of MRIP exhibited slower recovery rates and smaller mobile fractions. These cells form smaller clusters compared to DDR1bYFP and MRIP expressing cells cultured on collagen. Notably, the newly-formed clusters become disconnected from the cell bodies, which lead to even slower recovery rates (Figure 6D,E).

The contribution of DDR1 activation and MRIP to the formation of dense DDR1 clusters was studied with high spectral resolution, fluorescence resonance energy transfer (FRET). Cells were transfected with DDR1b-CFP (donor) and either DDR1b-YFP or the non-activating mutant DDR1b^K653A^-YFP (YFP^K653A^-acceptor) probes (Figure 6F). Representative donor and acceptor pre- and post-bleach images are shown for acceptor photobleaching-FRET (Figure 6G). De-quenched signals from the donor were evident after photo-bleaching the acceptor, demonstrating FRET and as a consequence, increased DDR1 clustering. Pre-bleach and post-bleach images were used to calculate mean FRET efficiencies. Cells expressing DDR1b-CFP and DDR1b-YFP probe cultured on collagen formed more numerous and denser DDR1 clusters compared with cells cultured on fibronectin, or cells that were null for MRIP that were transfected with the same probe (Figure 6H). Cell expressing DDR1b-CFP and DDR1b^K653A^-YFP probe exhibited 2-fold fewer and smaller DDR1 clusters compared with cells expressing DDR1b-CFP and DDR1b-YFP probe and cultured on collagen (Figure 6H). These data demonstrate that MRIP-dependent NMIA activity enables the formation of dense DDR1 clusters that are activated by autophosphorylation.

### 3.4. MRIP Dependent Contraction Cycle Regulates DDR1-NMIIA Association 

**Figure 7:**
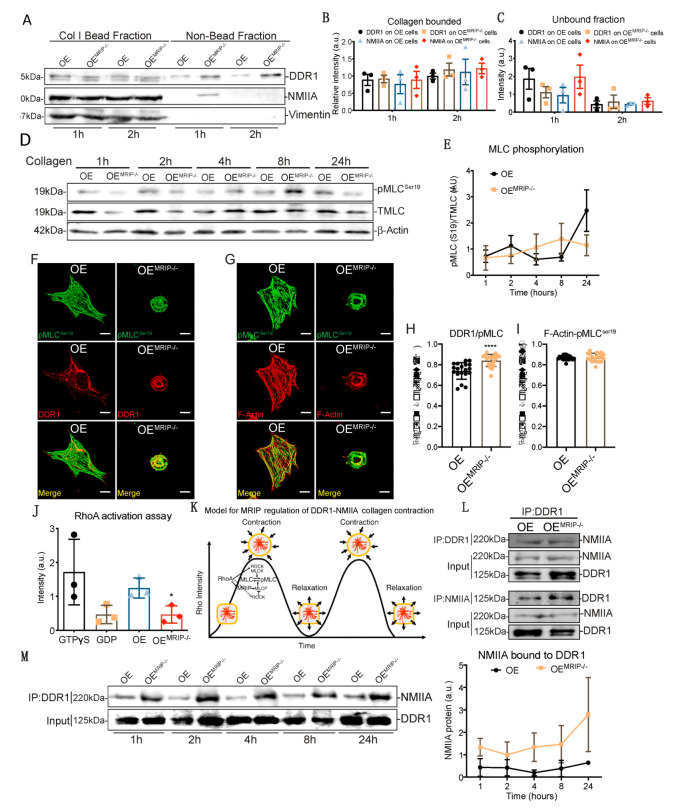
MRIP ablation enhances DDR1-NMIIA association but disrupts the myosin contraction cycle. (**A**) Collagen bead-bound fractions were prepared from OE and OE^MRIP-/-^ cells, which were immunoblotted for DDR1, NMIIA, and vimentin. Relative amounts of DDR1 and NMIIA in collagen- bound, (**B**) and unbound (**C**) fractions were quantified by densitometry of immunoblots, *n* = 3. (**D**), Whole-cell lysates of cells cultured on fibrillar collagen-coated tissue culture plates were immunoblotted for phospho-MLC^Ser19^ (#3675, CS), MLC #3672S, CS), and β-actin. (**E**) Quantification of pMLC^Ser19^/MLC by densitometry of immunoblots, *n* = 3. Cells were cultured on collagen for 3 h and co-immunostained for DDR1 (C-6: sc-374618) and pMLC^Ser19^ (#3671S, CS), (**F**), or for pMLC ^Ser19^ and F-actin, (**G**–**I**). Pearson coefficients were obtained by quantification of the fluorescent images using the colocalization2 plug-in in Fiji. *n* = 3, scale bar, 10 µm. (**J**) Cells were plated on collagen for 3 h, active GTP-RhoA was accessed by pull-down assay and measured by densitometry of GTP-RhoA/Total-RhoA on immunoblots, *n* = 3. (**K**) Temporal regulation of MRIP dependent myosin contractility. MRIP binds MLCP and RhoA and targets them to myosin enabling dephosphorylation of the MLC and consequently myosin contraction cycle. (**L**) DDR1 (C-6: sc-374618) or NMIIA (BT-564) was co-immunoprecipitated from cell lysates of cells cultured on fibrillar collagen. Immunoprecipitates were then immunoblotted for NMIIA or DDR1 respectively. (**M**) DDR1 (C-6: sc-374618) was immunoprecipitated from cell lysates of cells cultured on fibrillar collagen for indicated time points and immunoblotted for NMIIA. The immunoprecipitates were immunoblotted for NMIIA and DDR1, respectively. Quantification of NMIIA bound to DDR1 by densitometry of immunoblots, *n* = 2. Data are reported as mean ± SD for H and I or as mean ± SEM, *n* = 3. * *p* < 0.05, **** *p* < 0.0001.

GD25 OE and GD25 OE^MRIP-/-^ cells were cultured on tissue culture plastic (which does not activate DDR1). Fibrillar collagen-coated magnetite beads (~5 µm diameter) were then incubated on the dorsal surface of cells for one or two hours and the collagen bead-associated fraction was isolated magnetically as described [13] (Figure 7A). Similar amounts of DDR1 and NMIIA were detected in collagen adhesions prepared from both cell types, indicating that deletion of MRIP does not affect the recruitment of DDR1 and NMIIA to collagen adhesions (Figure 7A–C). However, there was a marked increase in NMIIA that was not bound to collagen in OE^MRIP-/-^ cells, indicating that after depletion of MRIP, a large amount of NMIIA was no longer recruited to collagen-DDR1 adhesions (Figure 7C).

MRIP is a scaffold protein that recruits RhoA to the MLCP-NMIIA complex [14]. This process thereby enables dephosphorylation of the MLC and consequently the initiation of the previously described myosin contraction cycle [15,16] (Figure 7K). We plated GD25 OE or OE^MRIP-/-^ cells on collagen for 24 h and evaluated pMLC^ser19^ over time (Figure 7D). Quantification of pMLC^ser19^/MLC ratios showed variations over time in cells expressing DDR1 and MRIP. In contrast, cells that were depleted of MRIP showed a steady, time-dependent activation of pMLC^ser19^ (Figure 7E). We also examined the colocalization of pMLC^ser19^ with DDR1 or actin filaments in GD25 OE and OE^MRIP-/-^ cells cultured on collagen. There was increased colocalization of pMLC ^ser19^ with DDR1 in OE^MRIP-/-^ cells compared with OE cells (Figure 7H). However, there were no marked differences of pMLC ^ser19^-F-actin colocalization between these two cell types (Figure 7I). These data indicate impaired recycling of phosphorylated NMIIA molecules into DDR1 adhesions of cells plated on collagen.

When plated on collagen, GD25 OE cells exhibited 2-fold higher RhoA activity than OE^MRIP-/-^ cells (Figure 7J)**.** In co-immunoprecipitation experiments, the DDR1-NMIIA association was not impacted by MRIP deletion (Figure 7L). However, when we analyzed the DDR1-NMIIA association over time (Figure 7M), this association was at a consistently high and stable level in MRIP null cells. In contrast, in cells expressing DDR1 and MRIP, the association between DDR1 and NMIIA varied in intensity, indicating a more dynamic and cyclic association. Taken together these data indicate that the MRIP-dependent NMIIA cycle is involved in the formation of DDR1 dense clusters. These clusters ultimately enable the transmission of NMIIA-dependent contractile forces to collagen fibrils.

## 4. Discussion

DDR1 is collagen mechanoreceptor aberrantly expressed in multiple epithelial solid cancer types. DDR1 expression is also increased in the stroma of invasive breast tumors [17,18] and in gastric cancer-associated fibroblasts that enhance peritoneal tumorigenesis [19]. We showed that through its associations with NMIIA, DDR1 links actomyosin contractility to increased collagen alignment and compaction. These alterations of collagen structure are seen in fibrotic lesions and in fibrotic tumor stroma [2,20]. As MRIP was enriched in collagen-DDR1-NMIIA complexes, we considered that MRIP regulates the kinetics of DDR1-NMIIA association that is required for collagen tractional remodeling. Our main findings are that: a) MRIP-dependent NMIIA activity was required for DDR1-dependent cell migration over collagen and collagen tractional remodeling; and b) MRIP-dependent NMIIA activity was required for the proper assembly and growth of DDR1 clusters on collagen. These clusters were sustainably activated by auto-phosphorylation.

**Figure 8:**
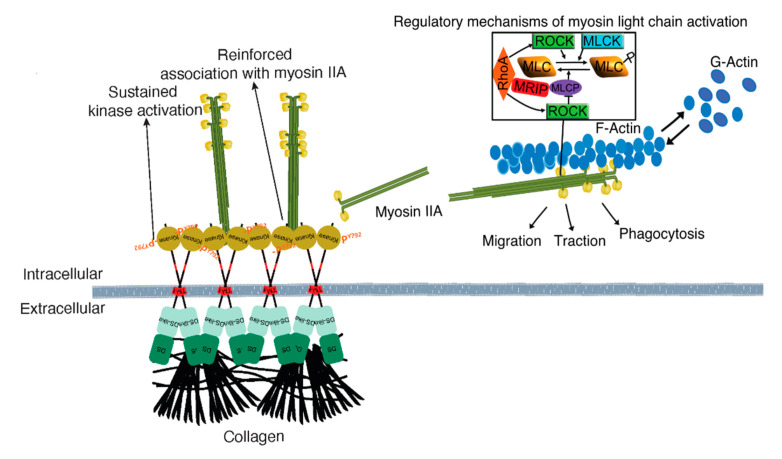
Mechanism of MRIP-dependent myosin IIA activity involved in DDR1 clustering and activation control collagen tractional remodeling. NMIIA filament assembly is mediated by myosin light chain kinase and ROCK, which enables DDR1-NMIIA association and force transmission to collagen [2]. DDR1 clustering and activation strengthen DDR1 binding to collagen and increase DDR1-NMIIA association and collagen tractional remodeling. Here we show that MRIP is a key regulator of the DDR1-NMIIA association. We show here that the association of DDR1 with NMIIA, the formation of DDR1 clusters, and the activation of DDR1 by autophosphorylation of Y792, requires MRIP. These processes ultimately determine the amplitude and kinetics of contractile force generation in the collagen-rich ECM.

Our previous data show that DDR1 binding to collagen leads to the association of the DDR1 C-terminal kinase domain with NMIIA filaments. Collagen-induced DDR1 clustering promotes activation of the kinase domain and enhances the association of DDR1 with NMIIA. This process generates a positive feedback loop that strengthens cell adhesion and optimizes the transmission of NMIIA-dependent contractile forces to collagen fibrils [2] (Figure 8). NMIIA is a motor protein involved in the generation of contractile forces required for directed cell migration and collagen remodeling [21,22]. The activity of NMIIA is controlled by the phosphorylation of the MLC, which is regulated by two groups of enzymes, MLC kinases and MLC phosphatases. NMIIA activity can also be regulated by RhoA via its downstream effector ROCK. In turn, ROCK can either phosphorylate the MLC directly or inhibit MLCP [23]. MRIP is a scaffold protein that binds RhoA, MLCP, and NMIIA and targets MLCP to dephosphorylate the MLC and enable a contraction cycle (Figure 8) [8,24]. We found that MRIP-dependent NMIIA activity facilitated the formation of DDR1 clusters. These clusters contribute to DDR1 activation by autophosphorylation and are involved in collagen tractional remodeling.

Cell migration involves the generation of membrane protrusions and cellular contraction [25]. MLC phosphorylation controls NMIIA contraction at the rear end of migrating cells while at the leading edge it is critical for control of membrane protrusions, actin retrograde flow, and focal adhesion turnover [15]. By mass spectrometry, immunoprecipitation, and immunostaining we found that, independent of β1 integrin, MRIP was enriched in DDR1 adhesions on collagen. These adhesions localized to the tip of cell extensions where MRIP colocalized with DDR1, NMIIA and F-actin. MRIP does not affect the activity of either MLCP or MLCK directly but rather it facilitates the access of MLCP to phosphorylation sites at the MLC to enable myosin dephosphorylation [8,14,24,26]. Inhibition of ROCK with Y27632 leads to disassembly of NMIIA filaments in the central part of the cell and consequently, cells move more rapidly [15]. MLCK inhibition leads to NMIIA filament disassembly at the cell periphery and as a consequence, cells migrate less efficiently [15,30]. Inhibition of MLCP blocks fibroblast migration, increases MLC phosphorylation, induces focal adhesion maturation, and promotes thickening of actin stress and cortical fibers [31]. We found that in cells depleted of β1 integrin, an integrin subunit present in all integrin adhesions that binds collagen, cell migration on collagen was facilitated by DDR1 and MRIP expression. Further, depletion of MRIP completely blocked cell migration over collagen. These data highlight the importance of MLCP in maintaining actomyosin-based cell function by regulating MLC phosphorylation. 

The hallmark of fibrotic diseases and the tumor-associated stroma is excessive, highly aligned, and extensively crosslinked collagen fibrils. The transmission of actomyosin contraction forces to collagen is mainly attributed to integrins and the activation of the Rho-ROCK pathway [32,33,34]. However, DDR1 expression and function are also strongly linked to fibrotic disorders such as atherosclerosis, arthritis, and several types of cancer [35,36,37,38]. Here we demonstrated that MRIP-dependent NMIIA activity was required for the generation of actomyosin contraction forces that are involved in DDR1-dependent collagen compaction, alignment, and contraction. Indeed, DDR1 overexpression enhances collagen compaction [39] and regulates collagen deposition and tissue architecture in the mouse auditory system [40]. Full-length, kinase active DDR1 is required for binding to MLCK-activated NMIIA filaments and for the force transmission involved in collagen tractional remodeling and mechanical remodeling [2]. Here we showed that MRIP regulation of MLC phosphorylation turnover at collagen adhesions was essential for collagen tractional remodeling.

DDR1 is an unusual RTK in part because it is a homodimer with slow but sustained kinetics of activation upon collagen binding [41,42]. The mechanism of DDR1 activation involves collagen-induced clustering of DDR1 dimers [2,10,11,43]. Here we found that cells expressing full-length DDR1 and MRIP and when cultured on collagen, formed more abundant and denser DDR1 clusters compared with cells cultured on fibronectin. Similar contrasts were seen with cells expressing DDR1 but null for MRIP, or cells expressing the non-activating DDR1 mutant (DDR1^K653A^-YFP). DDR1 oligomerization is required for high affinity binding to collagen [44]. We found previously that after stimulation with fibrillar collagen, pDDR1^Y792^ is mainly present as oligomers. These data support the notion that receptor oligomerization precedes receptor activation. Further, collagen induced accumulation of DDR1 into dense clusters, which leads to auto-phosphorylation of DDR1 at Y792 [10] and trans-phosphorylation of dimers on Y513 [11]. Accordingly, DDR1 receptor clustering induces DDR1 activation, which reinforces DDR1 binding to collagen, and initiates a feedforward mechanism that increases receptor clustering and enables collagen remodeling by traction [2]. We conclude that MRIP-dependent myosin activity was involved in the formation and growth of dense DDR1 clusters.

In this report we showed for the first time that MRIP depletion decreased DDR1 activation (Y792). DDR1 activation and myosin contraction increase when cells are plated on stiff collagen substrates or when cells cultured on soft collagen substrates are treated with LPA [2]. LPA increases intracellular tension by activating ROCK and inhibiting MLCP. We found that in cells depleted of MRIP, LPA-dependent ROCK activation rescued DDR1 clustering. The importance of the myosin cycle in supporting DDR1 clustering and consequent activation was further underlined by our results on inhibition of myosin motor activity by blebbistatin. This intervention was associated with reduced collagen-dependent DDR1 activation. These data indicate that NMIIA activity and its association with DDR1, are involved in mechanically supporting DDR1 cluster formation. These processes are also involved in their growth into dense clusters that support activation by autophosphorylation [10]. Cells expressing DDR1bYFP that were cultured on fibronectin, or cells expressing the non-activating mutant DDR1b^K653A^-YFP, showed diminished half-life recovery by FRAP and higher mobile fractions after photobleaching compared with cells expressing DDR1YFP and MRIP or cells depleted of MRIP. These findings support a previously suggested mechanism for DDR1 activation, which involves a feedforward loop between DDR1 activation and cluster growth [2,45]. In MRIP null cells, DDR1 clusters were smaller (quantified by immunofluorescence and FRET) and do not promote robust activation of DDR1, indicating that MRIP-dependent NMIIA activity is involved in enabling the feedforward loop between DDR1 clustering and activation.

NMIIA activity and filament assembly are controlled by the phosphorylation of the RLC on ser19 and thr18, by several kinases, most prominently by MLCK and by ROCK [46]. In contrast, only one single phosphatase, MLCP, dephosphorylates these sites. Further, the activity of the MLCP can be reduced by ROCK (Figure 8). We found that MRIP depletion did not change the recruitment of DDR1 and NMIIA to collagen adhesions and did not affect the colocalization of pMLC with F-Actin. However, MRIP depletion increased the amount of NMIIA in collagen-unbound fractions and the colocalization of DDR1 and pMLC^ser19^. Furthermore, MRIP deletion blocked the cyclic activation of pMLC^ser19^, reduced RhoA activation, and enhanced DDR1-NMIIA association at all time points. Because MRIP regulates RhoA activity and ROCK can also inactivate MLCP activity, it is expected that inactivation of RhoA by MRIP contributes to the increase in MLCP activity [26]. Here we found increased RhoA activation in MRIP-expressing cells, which is possibly explained by the different spatial and temporal regulation of RhoA. In smooth muscle cells, MRIP functions as a scaffold that links RhoA to the regulation of myosin phosphatase in stress fibers [24]. We also found that MRIP regulates MLC phosphorylation through interactions with RhoA, MLCP, or both, and enables the formation of mechanical functional DDR1 adhesions on collagen. MRIP depletion leads to a saturation of DDR1 adhesions with non-functional filamentous NMIIA that block the ability of cell to migrate and contract collagen.

In conclusion we show that MRIP is a key regulator of the association between DDR1 and myosin II. We demonstrate that the kinetics of this association supports DDR1 dense cluster formation and activation, both of which require MRIP. These processes ultimately determine the amplitude and kinetics of contractile forces that are applied to fibrillar collagen in the ECM. A more detailed understanding of these signaling pathways could suggest novel drug targets for clinical management of fibrotic diseases.
